# Protocol: A high-throughput DNA extraction system suitable for conifers

**DOI:** 10.1186/1746-4811-4-20

**Published:** 2008-08-01

**Authors:** Stanislav Bashalkhanov, Om P Rajora

**Affiliations:** 1 Faculty of Forestry and Environmental Management, University of New Brunswick, 28 Dineen Drive, Fredericton, New Brunswick, E3B 6C2, Canada

## Abstract

**Background:**

High throughput DNA isolation from plants is a major bottleneck for most studies requiring large sample sizes. A variety of protocols have been developed for DNA isolation from plants. However, many species, including conifers, have high contents of secondary metabolites that interfere with the extraction process or the subsequent analysis steps. Here, we describe a procedure for high-throughput DNA isolation from conifers.

**Results:**

We have developed a high-throughput DNA extraction protocol for conifers using an automated liquid handler and modifying the Qiagen MagAttract Plant Kit protocol. The modifications involve change to the buffer system and improving the protocol so that it almost doubles the number of samples processed per kit, which significantly reduces the overall costs. We describe two versions of the protocol: one for medium-throughput (MTP) and another for high-throughput (HTP) DNA isolation. The HTP version works from start to end in the industry-standard 96-well format, while the MTP version provides higher DNA yields per sample processed. We have successfully used the protocol for DNA extraction and genotyping of thousands of individuals of several spruce and a pine species.

**Conclusion:**

A high-throughput system for DNA extraction from conifer needles and seeds has been developed and validated. The quality of the isolated DNA was comparable with that obtained from two commonly used methods: the silica-spin column and the classic CTAB protocol. Our protocol provides a fully automatable and cost effective solution for processing large numbers of conifer samples.

## Introduction

Most genetic linkage mapping, marker-assisted selection, population and conservation genetic studies require processing of a large number of samples. Cost-effective high-throughput DNA extraction is a major bottleneck for many of these applications because handling and quantification of non-liquid samples, such as plant tissues, are labour-intensive and difficult to automate.

Traditional methods of DNA extraction involve multiple time-consuming steps, including organic solvent extraction and alcohol precipitation. Although commercially available spin column-based DNA preparation kits provide higher throughput, they are relatively expensive and difficult to automate. Recently-introduced magnetic fishing protocols allow for fully-automated, flexible throughput DNA isolation from certain samples, such as blood, but these methods are less tolerant to the secondary metabolites present in conifers and other plants [1]. Polysaccharides and phenolic compounds either impede DNA extraction or inhibit enzymatic reactions in the downstream applications. As a cheaper alternative to commercial DNA extraction kits, many labs use in-house developed simplified high throughput protocols, or use crude lysate as template for PCR [2,3]. However, majority of these protocols provide little quantitative information on the DNA yields, and were designed for crop species where the concentrations of PCR inhibitors are relatively low. Conifers require a more efficient DNA purification procedure. Although some protocols have been published for higher throughput DNA extraction from conifers [4,5], they still suffer from labour-intensive tissue grinding, high cost of silica columns or inconsistent DNA quality and yield.

Here we describe a cost-effective protocol based on the Qiagen MagAttract Plant DNA kit (QIAGEN Inc., Mississauga, Ontario) for high throughput DNA extraction from conifer needles and seeds. Most steps, including DNA quantification and normalization, can be done using an automated liquid handler.

## Materials

### Sample collection

DNA was extracted from silica-dried needles of red spruce (*Picea rubens*). Needles were collected in the field into plastic bags containing 5–10 g silica gel pouches as desiccant, and then stored in a freezer at -20°C upon arrival to the laboratory. With silica gel, the plant material could be kept at the ambient temperature for up to 14 days without degradation of DNA quality. The protocol was also tested and operationally used on seeds and fresh needles of red spruce and black spruce (*Picea mariana*), as well as needles of white spruce (*Picea glauca*) and eastern white pine (*Pinus strobus*).

### Reagents and consumables

The DNA isolation kit – Qiagen MagAttract Plant DNA Kit (Qiagen, Cat # 67161)

Lysis buffer – Qiagen AP1 buffer (Qiagen, Material # 1014630)

Ethanol

Disposable pipette tips

TE buffer, pH 7.5

2.0 ml microcentrifuge tubes, e.g. Sarstedt SafeLock tubes (Sarstedt, Cat # 72695)

Collection tubes in 96-well format racks (Qiagen, Cat # 19560)

Cap strips (Qiagen, Cat # 19566)

5 mm stainless steel balls (Glen Mills, Cat # 7400-004763-6)

Flat bottom 96-well microtiter plates (Greiner, Cat # 655101)

Round bottom 96-well microtiter plates (Greiner, Cat # 650101)

PCR plates (Greiner, Cat # 652270)

PicoGreen dsDNA quantification reagent (Invitrogen, Cat # P7581)

### Equipment

Laboratory balances with 0.001 g resolution

Multichannel pipette or an automated liquid handler

Mixer Mill MM300 (Retsch GmbH) or Qiagen TissueLyser mill

Water bath

Microcentrifuge

Centrifuge equipped with plate rotor, e.g. Eppendorf 5417 with the A-2-DWP-plate rotor or equivalent

96-well magnet, e.g. Qiagen Type B

Fluorescent plate reader or other instruments for measuring the DNA concentration

## Protocol

There are two versions of the protocol. Protocol A is designed for medium throughput and provides higher DNA yields per sample processed, whereas Protocol B works in the high-throughput 96-well format from the initial tissue homogenization to the purified DNA at the end.

### Protocol A – MTP version

1. Weigh approximately 150 mg of plant material in 2 ml microcentrifuge tubes

2. Add 500 μl of buffer AP1 and two 5 mm stainless steel beads. Grind the samples in a Mixer Mill for two rounds, 3 min each at 30 Hz

3. Incubate the samples at 65°C for 15 min

4. Clear the lysates by centrifugation at 18,000 g for 15 min

5. Transfer 170 μl of cleared lysate into a flat bottom 96-well plate

6. Add premixed 200 μl 98% ethanol and 15 μl MagAttract A suspension to lysate. Mix by pipetting and then incubate the mixture at the ambient temperature for 2 min. Mix by pipetting again and incubate for another 2 min

7. Place the plate onto the magnet and remove the supernatant from the sample

8. Resuspend the beads by pipetting in 200 μl of RPW buffer containing RNAse A as per the manufacturer's protocol

9. Place the plate onto the magnet and remove the supernatant from the sample

10. Resuspend the beads in 200 μl of 98% ethanol

11. Place the plate on the magnet and remove the supernatant, repeat 2 more times

12. Air dry the beads for 10 min

13. Resuspend the beads in 100 μl of 10 mM Tris-HCl pH 7.5, incubate 5 min at ambient temperature

14. Place the plate on the magnet and transfer the DNA into a 96-well PCR plate for storage. Normally 95–100 μl (DNA concentrations varied with species and are provided below) can be recovered.

NOTE: The original MagAttract buffer system didn't work well with conifers. The yield and quality of DNA were poor when original MagAttract buffers and protocol were used. The resulting DNA concentrations were less than 1 ng/μl and no PCR products could be obtained using these DNA extracts. We were able to overcome these problems by using AP1 buffer instead of RLT for lysis and ethanol instead of RB buffer for the DNA binding step.

We recommend the fluorimetric assay (e.g. PicoGreen by Invitrogen) as the best way to determine the DNA concentration in MagAttract-processed samples. Spectrophotometric (OD_260_) measurements in 96-well plates tend to give unstable results due to the possible sample-to-sample variation in the liquid meniscus shape which in turn leads to biased pathlength correction. The resulting DNA concentrations were 70 ± 15 ng/μl (~7 μg DNA/150 mg needle tissue) in *Picea rubens*, and 49 ± 13 ng/μl (~5 μg DNA/150 mg needle tissue) in *Picea glauca*. For subsequent genotyping, working plates containing 10 ng/μl DNA dilutions were prepared using an automated liquid handler.

### Protocol B – HTP version

1. Place 1–2 needles (3–5 mg dry weight) or one seed per sample in 96-racked collection tubes. Add 50 μl of buffer AP1 and one 5 mm stainless steel bead. Seal the tubes with cap strips

2. Grind the samples in a Mixer Mill for two rounds, 3 min each at 30 Hz

3. Briefly spin the racked tubes in a plate rotor at 2000 g and add another 150 μl of buffer AP1. Re-cap the tubes and mix the contents by shaking

4. Incubate the samples at 65°C for 15 min

5. Clear the lysates by centrifugation at 2200 g for 10 min in a plate rotor

6. Transfer 85 μl of cleared lysate into a round bottom 96-well plate

7. Add premixed 130 μl 98% ethanol and 12 μl MagAttract A suspension. Mix by pipetting. Incubate at the ambient temperature for 2 min. Mix by pipetting again and incubate for another 2 min

8. Place the plate onto the magnet and remove the supernatant from the sample

9. Resuspend the beads by pipetting in 150 μl of RPW buffer containing RNAse A as per the manufacturer's protocol

10. Place the plate onto the magnet and remove the supernatant

11. Resuspend the beads in 200 μl of 98% ethanol

12. Place the plate on the magnet and remove the supernatant, repeat 2 more times

13. Air dry the beads for 10 min

14. Resuspend the beads in 130 μl of 10 mM Tris-HCl pH 7.5, incubate 5 min at ambient temperature

15. Place the plate on the magnet and transfer the eluted DNA into a 96-well PCR plate for storage. Normally 125–130 μl (DNA concentrations varied with species and tissues and are provided below) can be recovered.

NOTE: We have eliminated the weighing step as one of the most time-consuming operations by using a few needles instead. Depending on the foliage size and shape, the amount of the plant material should be adjusted at the beginning to keep the dry weight of sample around 3–5 mg. The resulting DNA concentrations varied from 5.6 ± 2.0 ng/μl (~1 μg DNA/3–5 mg needle tissues) for *Picea rubens *to 9.7 ± 6.4 ng/μl for *Pinus strobus*, according to the fluorimetric PicoGreen assay. For *Picea rubens *and *Picea mariana *seeds, the DNA concentrations were 28.3 ± 13.8 ng/μl (~3.5 μg DNA/seed) and 6.2 ± 1.8 ng/μl (~0.8 μg DNA/seed), respectively. Seeds were processed as they the came from the storage without additional manipulations except for the visual inspection. Seed coats were not removed. 1.0–1.2 μl of eluates were used as templates for PCR.

## Comments

**Figure 1:**
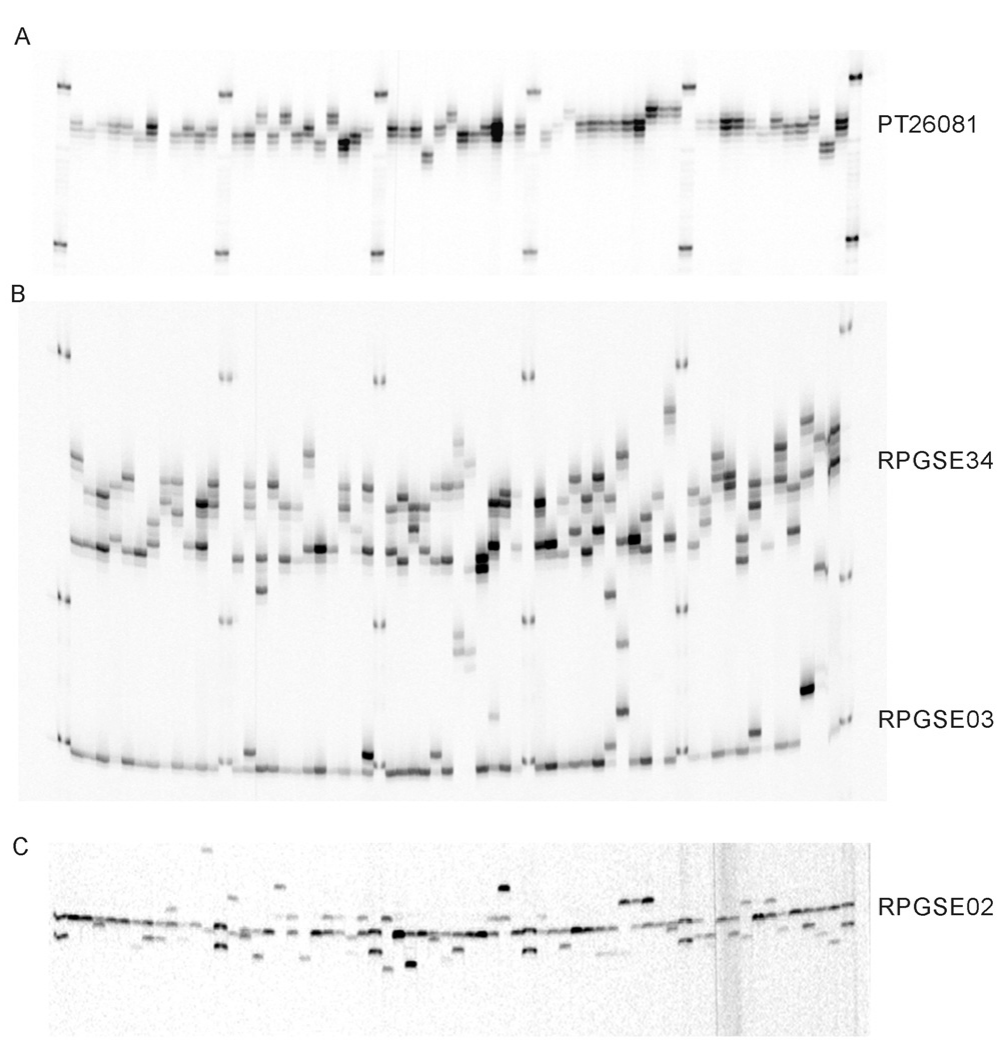
**Microsatellite genotyping of 60 spruce trees with SSRs containing mono-, di-, and trinucleotide repeats**. **A**: Gel image of fragment patterns revealed by mononucleotide chloroplast-encoded microsatellite PT26081 in *Picea rubens *[7]. **B**: Gel image of fragment patterns revealed by dinucleotide repeat (RPGSE34) and trinucleotide repeat (RPGSE03) nuclear microsatellite loci in *Picea rubens*. The PCR was tetraplex with two other loci being genotyped in the second channel. **C**: Gel image of fragment patterns revealed by trinucleotide repeat (RPGSE02) nuclear microsatellite locus in *Picea glauca*.

We validated the performance of the resulting DNA preparations by multiplex PCR assays. Amplicons of expected size were obtained. Later on more than 3000 needle and seed samples of *Picea rubens, Picea glauca*, and *Picea mariana *were processed and genotyped in our lab using more than a dozen nuclear and chloroplast microsatellites (Figure 1). Reproducible assays were possible with 4-plex reactions. DNA isolation for one 96-well plate takes approximately 3.5 hours start to finish, and the use of a robotic liquid handler further reduces the hands-on time to about 40 min.

### Comparison with other DNA extraction methods

To determine the reproducibility and efficiency of DNA isolation, we have compared our Protocol A-MTP with a slightly modified protocol using Qiagen DNeasy spin columns (Qiagen), and a version of widely used CTAB method [6]. Since some variability in the efficiency of mechanical tissue disruption is unavoidable due to the natural heterogeneity of plant material, we performed parallel DNA extractions using all three protocols in 8 replicates from the same bulked homogenized sample. Twenty grams of spruce needles were ground into fine powder under liquid nitrogen using mortar and pestle. 150 ± 2 mg of ground tissue were transferred into 2 ml Eppendorf tubes along with two 5 mm stainless steel balls normally used for tissue grinding. Then the DNA isolation was performed as follows:

#### Modified MagAttract protocol

As outlined above in the section Protocol A.

#### CTAB

The plant material was resuspended in 900 μl of 2× CTAB buffer (2% cetyltrimethylammonium bromide (CTAB); 1.4 M NaCl; 100 mM Tris-HCl, pH 8.0; 20 mM EDTA; 1% PVP; 0.2% β-mercaptoethanol) and 4 μl RNAse A (4 mg/ml, Qiagen) for 1 min, then incubated for 40 min at 65°C. The lysates were extracted twice with equal volume of chlorophorm:isoamyl alcohol (24:1). DNA was precipitated with 0.7 volume of 100% isopropanol (30 min at -20°C, followed by centrifugation at 18000 g for 10 min). The DNA pellet was washed with cold 70% ethanol twice, vacuum dried, and resuspended in 100 μl of TE buffer.

#### Qiagen DNeasy

DNA was isolated according to the manufacturer's instructions, except for increased volumes of AP1 and AP2 buffers (700 μl and 250 μl per prep, respectively). Purified DNA was eluted into 150 μl of 10 mM Tris-HCl pH 7.5.

**Figure 2:**
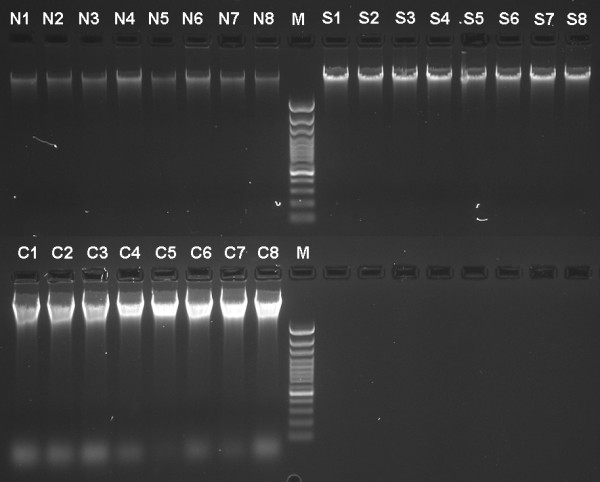
**Comparison of DNA yield and quality across different isolation methods**. Genomic DNA extracted from red spruce needles using our MagAttract-based protocol (N1–N8), DNeasy spin columns (S1–S8), and CTAB protocol (C1–C8). M – GeneRuler 100 bp DNA Ladder Plus (MBI Fermentas). Five microliters of each sample were loaded onto 1.5% agarose gel containing SYBR Safe DNA Gel Stain (Invitrogen).

All three methods resulted in high quality DNA (Figure 2) with A260/280 values in the range 1.69–1.85. The DNA yield varied greatly among the methods (Table 1). Significant variance of DNA concentration in our protocol can be attributed to relatively high pipetting errors when handling magnetic particles.

**Table 1:** Comparison of MagAttract-based protocol with Qiagen DNeasy spin columns and CTAB protocol.

Method		DNA concentration, ng/μl	DNA yield, μg/preparation	CV, %
MagAttract-based protocol	OD_260_	75	7.5	30.66
	fluorimetric	70	7.0	37.98
DNeasy	OD_260_	121	18.1	9.59
	fluorimetric	190	28.5	10.73
CTAB	OD_260_	875	87.5	8.10
	fluorimetric	1079	107.9	13.12

## Conclusion

The CTAB method demonstrated the best yield and good reproducibility and probably remains the method of choice where large amounts of high quality DNA are required. For research projects requiring processing of larger sample sizes, our MagAttract-based protocol can be a flexible and cost-efficient solution (around $1 per sample, compared to $4 for spin columns). Medium-throughput version of our protocol allows for processing of large sample sets while maintaining higher DNA yield. The high-throughput variation is the best solution for genotyping projects where speed is the ultimate priority. It allows for processing up to 200 samples per person per day, in comparison to other protocols where expected output would be around 40–60 samples. The protocol works from start to end in the standard SBS 96 well microplate format and is fully automatable.

## Competing interests

The authors declare that they have no competing interests.

## Authors' contributions

SB carried out the laboratory work, developed the protocol and designed the robotic liquid handler software methods. OPR is the principal investigator of the project. He supervised the research work and provided funding and direction. Both authors participated in writing and revising the article, and approved the final manuscript.
